# Supplementary material for: Simple Self-Assembly Strategy of Nanospheres on 3D Substrate and Its Application for Enhanced Textured Silicon Solar Cell
Source: Nanomaterials (Basel). 2021 Sep 30;11(10):2581. doi: 10.3390/nano11102581 (PMC8541415; doi:10.3390/nano11102581)
Supplement: Supplementary file 1 [file nanomaterials-11-02581-s001.zip › nanomaterials-1355995-supplementary.pdf]

nanomaterials

Supplementary material

Simple Self-Assembly Strategy of Nanospheres on 3D Substrate and Its Application for Enhanced Textured Silicon Solar Cell

Dan Su [1,†], Lei Lv [2,†], Yi Yang [2], Huan-Li Zhou [2], Sami Iqbal [2] and Tong Zhang [1,2,3,*]

[1] Key Laboratory of Micro-Inertial Instrument and Advanced Navigation Technology, Ministry of Education, School of Instrument Science and Engineering, Southeast University, Nanjing 210096, China; jssysls@163.com
[2] Joint International Research Laboratory of Information Display and Visualization, School of Electronic Science and Engineering, Southeast University, Nanjing 210096, China; lvlei@seu.edu.cn (L.L.); yyang19x@163.com (Y.Y.); huanli_zhou@163.com (H.-L.Z.); 101300112@seu.edu.cn (S.I.)
[3] Suzhou Key Laboratory of Metal Nano-Optoelectronic Technology, Southeast University Suzhou Campus, Suzhou 215123, China
* Correspondence: tzhang@seu.edu.cn
† Equally contributed.

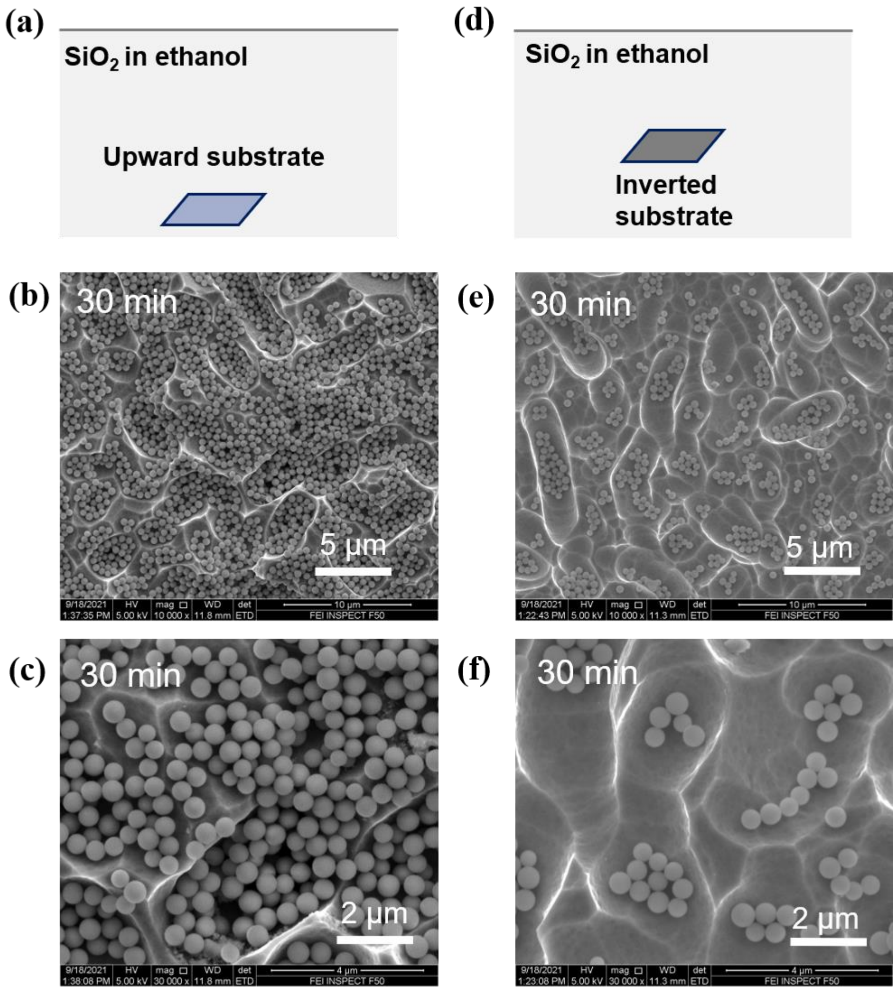

Figure S1. Self-assembly nanocoating under different substrate orientations in the SiO_2 assembly colloid with an assembly time of 30 min. (**a**)-(**c**) Schematic illustration and SEM graphs of the assembly with an upward substrate, (**d**)-(**f**) Schematic illustration and SEM graphs of the assembly with an inverted substrate.

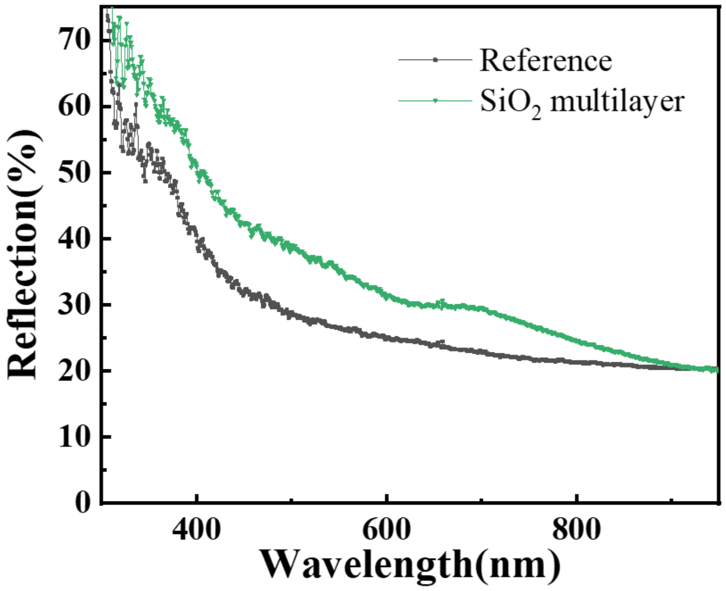

Figure S2. Optical reflection measurement of the textured silicon solar before (black line) and after (green line) five-round SiO_2 repeated assembly process.

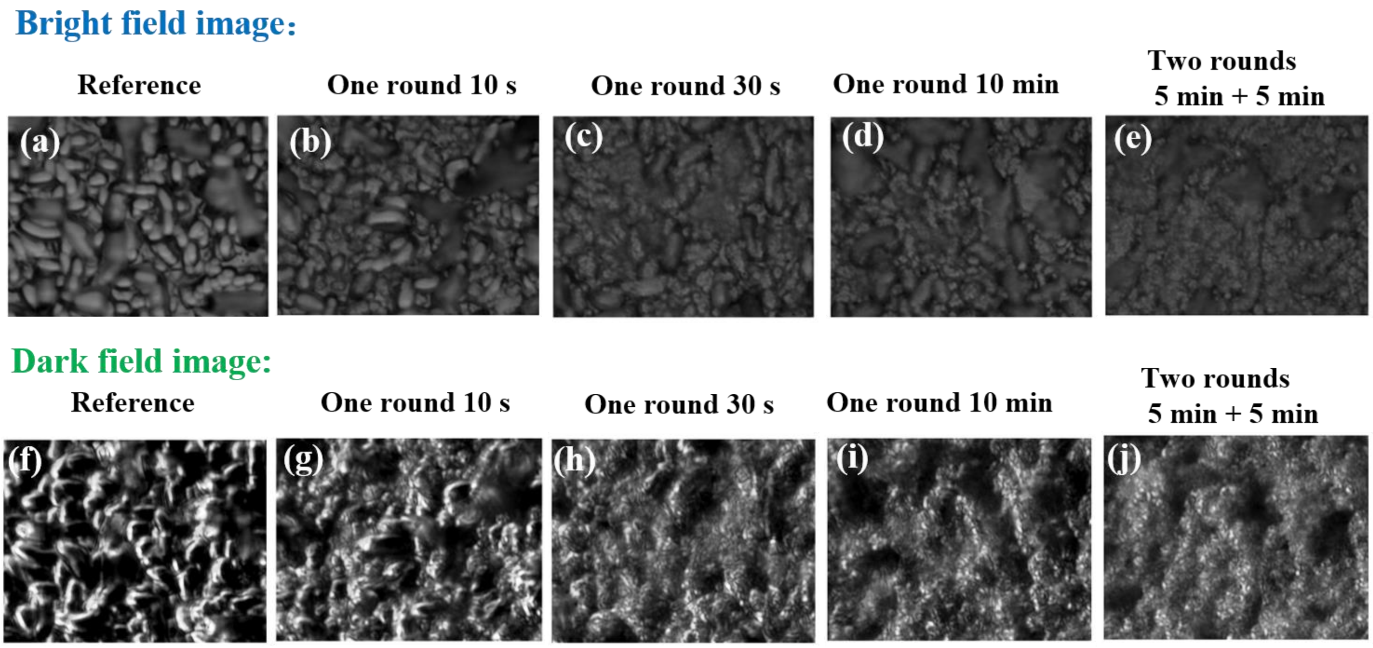

Figure S3. Grayscale image transformation of the micro-region optical images. Bright filed grayscale image for (**a**) reference solar cell, (**b**) after one round 10 s assembly, (**c**)after one round 30 s assembly, (**d**) after one round 10 min assembly, and (**e**) after two round 5 min+5 min assembly. Dark filed grayscale image for (**f**) reference solar cell, (**g**) after one round 10 s assembly, (**h**)after one round 30 s assembly, (**i**) after one round 10 min assembly, and (**j**) after two round 5 min+5 min assembly.

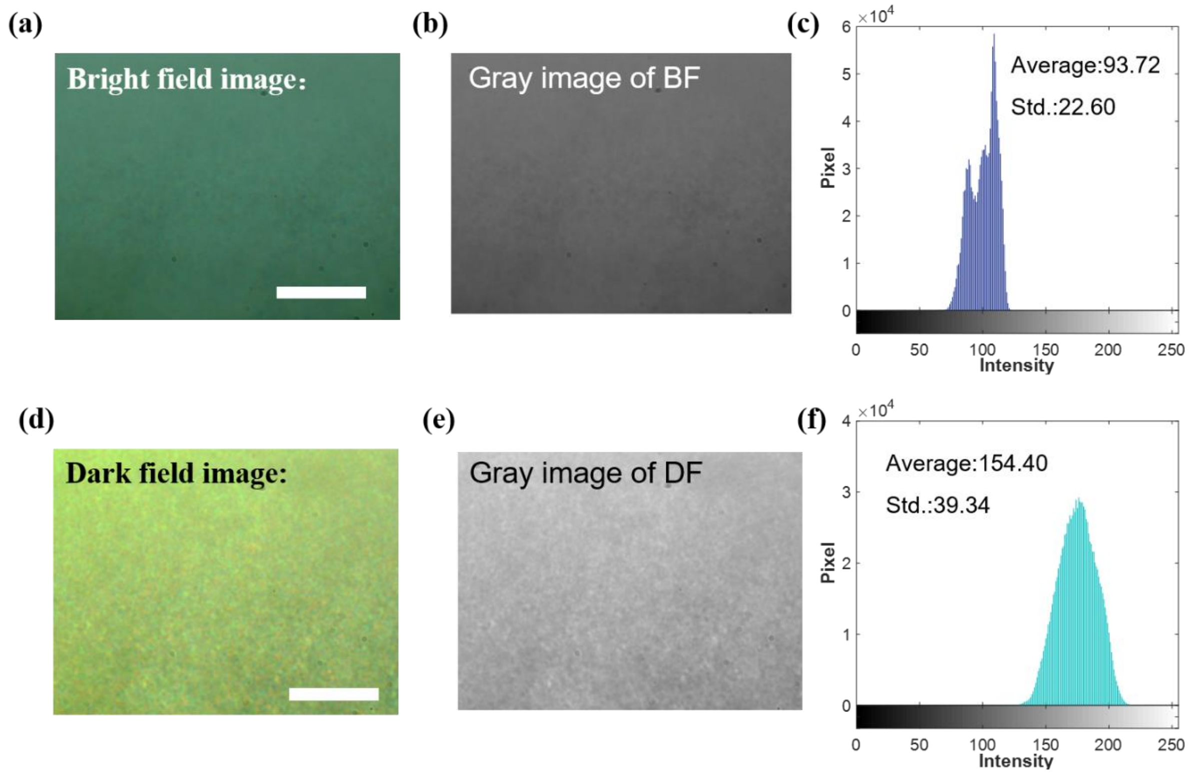

Figure S4. Micro-region optical image analysis of solar cell after five-round SiO_2 repeated assembly process. (**a**) Original bright field image, (**b**) Bright field gray image after grayscale transformation, (**c**) Intensity distribution histogram of bright field gray image, (**d**) Original dark field image, (**e**) Dark field gray image after grayscale transformation, (**f**) Intensity distribution histogram of dark field gray image.
